# On the Al–Al_11_Ce_3_ Eutectic Transformation in Aluminum–Cerium Binary Alloys

**DOI:** 10.3390/ma13204549

**Published:** 2020-10-13

**Authors:** Frank Czerwinski, Babak Shalchi Amirkhiz

**Affiliations:** CanmetMATERIALS, Natural Resources Canada, Hamilton, ON L8P 0A5, Canada; Babak.Shalchi_Amirkhiz@Canada.ca

**Keywords:** aluminum–cerium alloys, eutectic, solidification, rare earths

## Abstract

The L ↔ Al + Al_11_Ce_3_ technologically important eutectic transformation in Al–Ce binary alloys, containing from 5 to 20 wt.% Ce and ranging from hypo- to hypereutectic compositions, was examined along with the microstructure and properties of its solidified product. A combination of thermal analysis and metallography determined the coordinates of the eutectic point at 644.5 ± 0.6 °C and 10.6 wt.% Ce, clarifying the existing literature ambiguity. Despite the high entropy of melting of the Al_11_Ce_3_ phase, in hypoeutectic alloys the eutectic was dominated by the regular morphology of periodically arranged lamellae, typical for non-faceted systems. In the lamellar eutectic, however, the faceting of Al_11_Ce_3_ was identified at the atomic scale. In contrast, for hypereutectic compositions, the Al_11_Ce_3_ eutectic phase exhibited complex morphology, influenced by the proeutectic Al_11_Ce_3_ phase. The Al_11_Ce_3_ eutectic phase lost its coherency with Al; it was deduced that a partial coherency was present only at early stages of lamellae growth. The orientation relationships between the Al_11_Ce_3_ and Al in the eutectic structure, leading to partial coherency, were determined to be [0 0 1]_Al_ ║ $\left[\bar{1}\;1\;1\right]$_Al11Ce3_ with $\left(0\;4\;\bar{4}\right)$_Al_ ║ $\left(\bar{2}\;0\;0\right)$_Al11Ce3_ and [0 1 1]_Al_ ║ $\left[\bar{3}\;0\;1\right]$_Al11Ce3_ with $\left(\bar{2}\;0\;0\right)$_Al_ ║ (0 6 0)_Al11Ce3_. The Al_11_Ce_3_ phase with a hardness of 350 HV and Al matrix having 35 HV in their eutectic arrangement formed in situ composite, with the former playing a role of reinforcement. However, the coarse and mostly incoherent Al_11_Ce_3_ eutectic phase provided limited strengthening and the Al–Ce alloy consisting of 100% eutectic reached at room temperature a yield stress of just about 70 MPa.

## 1. Introduction

Aluminum alloys, due to a combination of their light weight, high strength, and ductility, have been proven to be attractive structural materials in modern age applications. The detrimental feature of the presently available grades is their strengthening mechanism, which becomes ineffective at increased temperatures, required by advanced automotive and aerospace designs, as controlling phases rapidly coarsen and dissolve [1]. Since in cast Al alloys, eutectic is the essential component that provides the necessary fluidity in the liquid state, to achieve a high thermal stability the solidified eutectic structure should remain stable at service temperatures. 

In conventional cast Al–Si alloys, the high temperature mechanical performance may be enhanced by alloying with transition metals, including Ti, Zr, V, Mo, Mn or Cr [2]. Having diffusion coefficients in Al lower by three to four orders of magnitude than those of Si or Mg, they form coarsening-resistant precipitates. However, despite thermally stable precipitates, the Al–Si eutectic imposes a limit on the alloy stability improvement. To overcome this barrier, the Al–Al_3_Ni eutectic was introduced [3,4] with better thermal stability, attributed to the diffusion coefficient of Ni in Al, being lower by about two orders of magnitude and higher eutectic temperature [5]. A possibility of further improvement is anticipated through a substitution of nickel with rare-earth metal cerium [6,7], having the diffusion coefficient in Al lower by approximately four orders of magnitude than that for Ni in Al [8].

**Figure 1:**
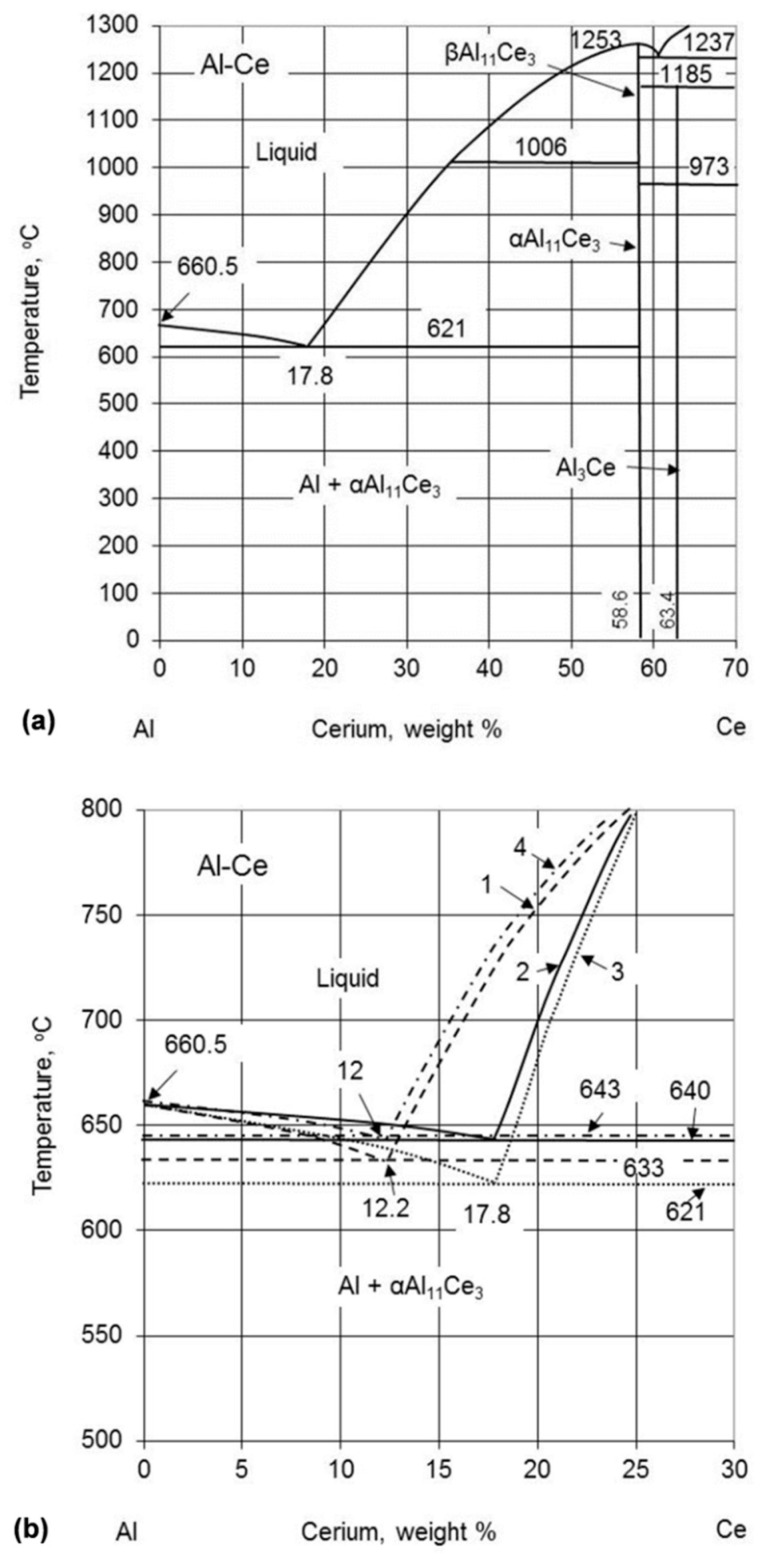
The Al rich portion of the Al–Ce phase diagram by Okamoto, 2011 [11] (**a**) and examples of the literature differences in the coordinates of the eutectic point (1) [12] 1990, (2) [13] 1998, (3) [11] 2011, (4) [14] 2017 (**b**). Values of the coordinates of the eutectic point, published within the last three decades, are listed in Table 1. Figure 1a was adapted from [11].

Although the idea of using cerium for aluminum alloying, with contents reaching eutectic compositions, was introduced over a century ago [9,10], there are still no commercial alloys with large-scale applications. The high-order phase diagrams Al–Ce–X_1…n_ are scarce and there is an ambiguity surrounding the Al–Ce binary system (Figure 1a). This includes the technologically important eutectic on the Al-rich side, Al + Al_11_Ce_3_, with discrepancies in the eutectic point coordinates exceeding 20 °C and 10 wt.% of Ce (Figure 1b and Table 1).

This paper describes the L ↔ Al + Al_11_Ce_3_ eutectic transformation in the Al–Ce binary alloys within a wide range of Ce contents along with morphology, crystallography, and properties of the solidified product. These data are needed to establish fundamentals for the development of multi-component light weight aluminum alloys that explore the Al–Ce eutectic.

## 2. Material Synthesis and Experimental Details

### 2.1. Alloy Casting

The Al–Ce alloys with a nominal Ce content from 5 wt.% to 20 wt.% were cast in our experimental foundry, using a clay–graphite crucible coupled with a resistance furnace under a protective atmosphere of argon. To determine the eutectic coordinates an increased number of alloys was cast around the suspected eutectic composition. The charge makeup with a weight of 5 kg was comprised of elemental Al and Ce, both of 99.9 wt.% purity. After melting, a liquid alloy was held at 780–800 °C for 0.5 h under intense mixing, followed by measurements and adjustments of the chemical composition. Then, the dross/oxide was removed from the melt surface and the crucible content was poured at 730 °C into multiply steel molds with an internal diameter of 32 mm and a height of 127 mm, coated with boron nitride. Following solidification, the cerium concentration was determined by inductively coupled plasma (ICP) method in accordance with MCLM F-23C and BAERD-GEN-018E specifications. The contents of the remaining elements were measured by optical emission spectroscopy (OES). Detailed chemical compositions are listed in Table 2.

### 2.2. Thermal Analysis

An investigation of the phase nucleation and growth during solidification was conducted using a computer-aided cooling curves thermal analysis (CA-CCTA) associated with measurements by Universal Metallurgical Simulator and Analyzer (UMSA) [24]. The system employs cylindrical samples with a diameter of 31 mm and a height of 35 mm, having a pre-drilled hole with an insert of stainless steel tube for a thermocouple. The test samples were processed in low thermal mass stainless steel foil, coated with boron nitride and protected against oxidation in the UMSA chamber with an inert argon atmosphere. Controlled heating to 780 °C was performed at a rate of 0.4 °C/s, followed by isothermal holding at 780 °C for 5 min and natural cooling to 50 °C at a rate of 0.2 °C/s. Since 780 °C was found to be insufficient for hypereutectic alloys, these chemistries were re-run using 850 °C as the maximum temperature for the UMSA furnace setting. For each composition, three heating/cooling cycles were completed to verify a repeatability of readings. 

### 2.3. Structural Analysis

The phase composition was measured using the Bruker D8 Discovery with a DaVinci Design X-ray diffractometer, equipped with a cobalt-sealed tube source (λCoα1 = 1.78886 Å/λCoα2 = 1.79277 Å (λ_avg_ = 1.79026 Å). During the testing, 2D frames were collected with DIFFRAC Measurement Centre Version 4.0 software and integrated to 1D using DIFFRAC EVA Version 4.0. Metallographic samples were prepared using the conventional surface preparation process starting from grinding to polishing. No etching was applied and the as-polished surfaces were examined with an optical microscope Olympus BX534 and scanning electron microscope FEI Nova NanoSEM650. The transmission electron microscopy was conducted using FEI’s Tecnai Osiris TEM equipped with X-FEG gun at 200 keV. Specimens for TEM were prepared by grinding to a thickness of 100 µm, cutting discs with a diameter of 3 mm and ion milling. Conventional bright field (BF), selected area electron diffraction (SAD), convergent beam electron diffraction (CBED) techniques were used for phase identification and crystallography assessment. STEM mode with bright field (BF) and high angle annular dark field (HAADF) detectors were employed in combination with energy dispersive spectrometry (EDS). The EDS with the ESPRIT software, applying a deconvolution of overlapping peaks was used for mapping the elemental distribution. Due to the Cu grid used in a sample holder its presence may be seen in the EDS background. High resolution transmission electron microscopy (HRTEM) examinations were performed at 400 kV with a point-to-point resolution of 0.16 nm. Based on the HRTEM images, the Fast Fourier transform (FFT) and inverse fast Fourier transform (IFFT) images were generated. Electron diffraction modelling was performed with the CrystalMaker^®^ software.

### 2.4. Mechanical Properties

The effect of Ce/eutectic on alloy strengthening was assessed through the room temperature uniaxial compression test, according to the ASTM E4 standard. The cylindrical samples with a diameter of 5 mm and a length of 8 mm were cut from the as-cast state with the same orientation along the mold axis. The compression test was performed at room temperature using the BÄHR DIL 805 deformation dilatometer for maximum strain of ε = 0.1 and a strain rate of 0.005 1/s. Microhardness was tested using the Clemex with a load of 200 gf to measure an average alloy hardness and 20 gf to measure the hardness of individual phases.

## 3. Results

### 3.1. Thermal Events during Alloy Solidification

The as-cast structures often exhibit the macro-segregation of alloying and impurity elements, accompanied by gas and shrinkage porosity that affect the solidification thermal output. It was assumed, therefore, that for cast alloys, the UMSA technique, through employing a relatively large sample of over 25 cm^3^, corresponding to about 70 g of Al alloy, was more appropriate than DTA/DSC techniques that use micro-size samples [24]. For this reason, the computer-aided cooling curves thermal analysis was selected as the main tool to determine the thermal events taking place during the solidification of Al–Ce alloys over a wide range of Ce contents. Since a cooling curve represents the balance between the evolution of heat in the sample and the heat flow away from the sample, the beginning of the solidification can be determined by the latent heat associated with a liquid–solid transformation. Some reactions, however, do not release enough latent heat to be distinguished on cooling curves and to improve the detection accuracy of thermal events, the derivatives of cooling curves dT/dt were calculated. 

**Figure 2:**
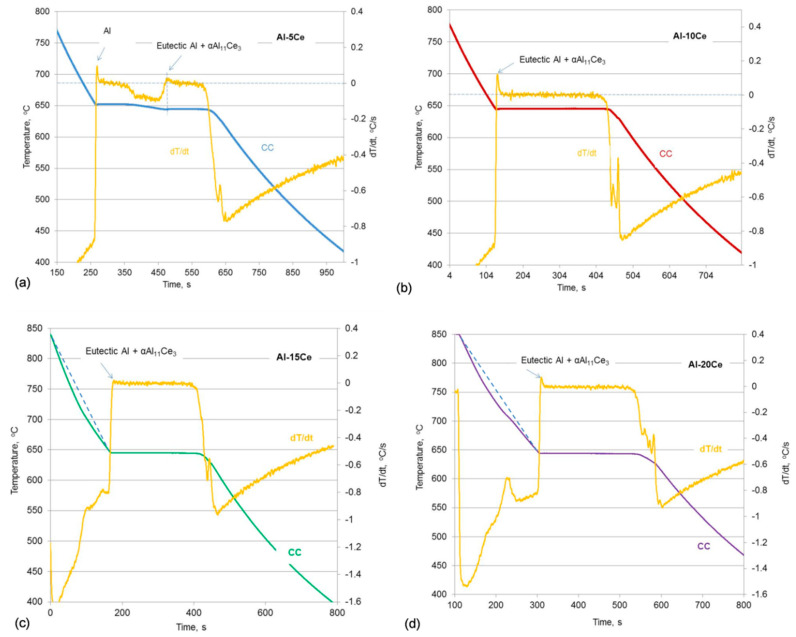
Computer-aided cooling curves’ thermal analysis showing the cooling curves (CCs) and the first derivative curves (dT/dt) plotted versus time with major thermal events recorded for Al–Ce alloys: (**a**) Al–5Ce; (**b**) Al–10Ce; (**c**) Al–15Ce; (**d**) Al–20Ce.

The Al–5Ce alloy exhibited hypoeutectic characteristics, where the first peak of dT/dt, aligned with a change of the slope on cooling curve, was associated with the beginning of solidification of the proeutectic Al phase (Figure 2a). The following distinct peak of derivative dT/dt, aligned with a beginning of the plateu on cooling curve, indicates the eutectic reaction. A difference between both temperatures of about 5 °C reveals the very narrow gap between the liquidus and solidus (eutectic) for the hypoeutectic Al–5Ce composition. The Al–10Ce alloy with the exact Ce content of 10.61 wt.% revealed exclusively the eutectic reaction (Figure 2b). A smooth cooling curve with a distinct plateau did not show features of proeutectic solidification of either Al or Al_11_Ce_3_. Increasing the Ce content to 11 wt.%, 12 wt.% and even 15 wt.% did not lead to noticable peaks on the cooling curves. As shown in Figure 2c for 15 wt.% Ce, the temperature versus time plots were dominated by a distinct plateau related to the eutectic transformation. However, the first derivative curve dT/dt exhibited peaks, preceeding the plateau for cooling times below 200 s. In this respect, a closer look at a cooling curve indicated a change in the slope that correlated with the peaks’ location on the first derivative curve. There is a similar scenario for the Al–20Ce alloy, where the first derivative revealed an enlarged peak for cooling times of 200–300 s, located at substantially higher temperatures, than the eutectic one (Figure 2d). Again, the portion of the cooling curve, corresponding to peaks on the first derivative showed a change in the slope, apparently related to the proeutectic solidification. To better show the slope change on the cooling curves for the Al–15Ce and Al–20Ce alloys, straight lines were added above the eutectic transformation. 

**Figure 3:**
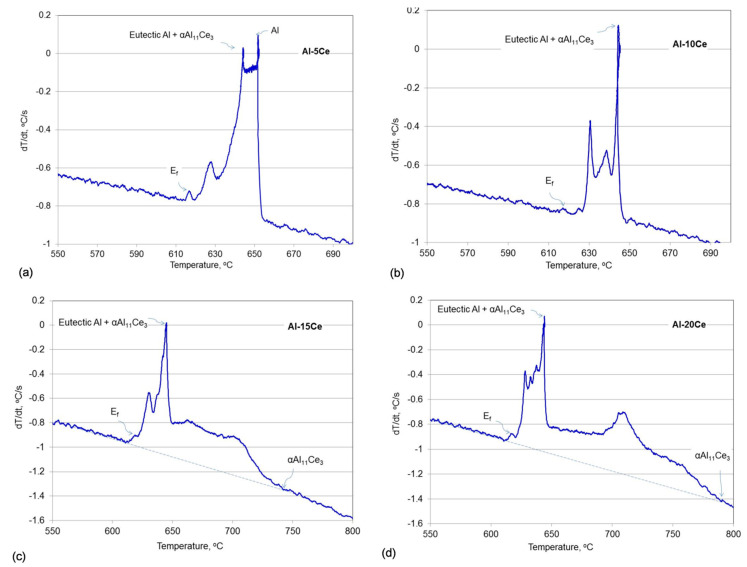
First derivatives of the cooling curves (dT/dt) plotted versus temperature (T) for Al–Ce alloys with marked proeutectic solidification of Al, Al_11_Ce_3_ and eutectic reactions in alloys investigated: (**a**) Al–5Ce; (**b**) Al–10Ce; (**c**) Al–15Ce; (**d**) Al–20Ce. E_f_—end of the non-equilibrium eutectic solidification.

When assessing the first derivative curves, in addition to the features related to proeutectic solidificataion, there were also evident peaks, located at the transient between the eutectic plateau and the temperature drop on the cooling curves that followed. These peaks were present for all alloy compositions (Figure 2a–d). To correlate the above named peaks with specific thermal events and determine their temperatures, the first derivatives were plotted versus the temperature. As seen in Figure 3a–d, peaks corresponding to eutectic temperatures, where dT/dt exceeds 0, are followed by peaks of lower intensity with the final one marked as E_f_. For all the alloy compositions, peaks E_f_ are located at 617–618 °C, i.e., over 25 °C below the eutectic temperature. It was concluded that the peak E_f_ indicates the temperature of the end of the non-equilibrium eutectic solidification. The liquidus temperatures for the hypereutectic alloys, describing the nucleation of the primary Al_11_Ce_3_ phase is determined in Figure 3c,d. Due to a substantial enlargement of the peaks above the background level, to determine the nucleation temperature of the proeutectic Al_11_Ce_3_, the base line was plotted. The determined temperatures of all thermal events, recorded for three separate experiments along with their statistical assessment, are summarized in Table 3.

### 3.2. Solidification Sequence–Solid Fraction versus Temperature Plot

**Figure 4:**
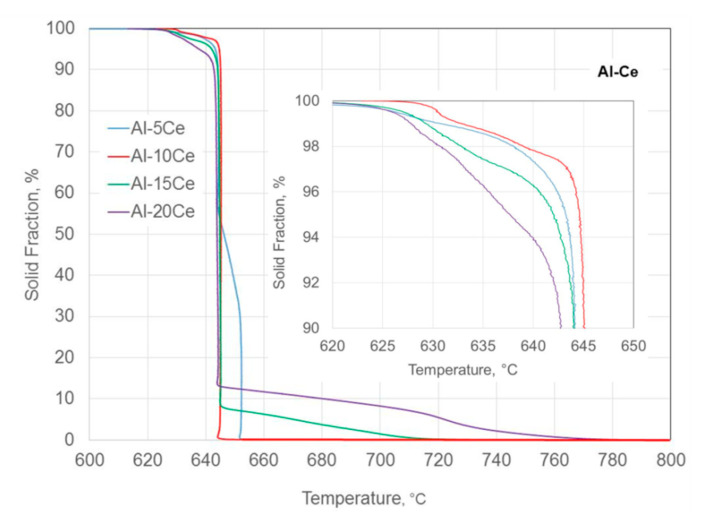
Experimental solid fraction versus temperature, determined based on the cooling curves, measured in the UMSA apparatus. The inset shows the non-equilibrium solidification of the small liquid fraction below the eutectic temperature.

The solid fraction versus temperature plot, obtained from cooling curves through the UMSA software, is shown in Figure 4. When combined with cooling and first derivative curves, this allowed to determine the solidification sequence of all individual alloys. 

For the Al–5Ce hypoeutectic composition, the solidification started from the nucleation of the proeutectic Al and continuued until the solid fraction reached about 52%. Then, the eutectic growth began and continued isothermally until the solid fraction reached about 96%. The remaining liquid fraction of 4% solidified under non-equilibrium conditions when the temperature decreased to around 617–618 °C. For the Al–10Ce eutectic composition, the liquid fraction subjected to non-equilibrium solidification was the smallest one of just above 2%. The scenario changed for hypereutectic compositions. In the case of the Al–15Ce and Al–20Ce alloys, about 8% and 13% liquid fraction, respectively, was transformed to the proeutectic Al_11_Ce_3_ phase, before the euteectic temperature was reached and the eutectic transformation could proceed. For these alloys, the liquid fraction subjected to non-equilibrium solidification was higher, reaching 4%–8% and increased with the Ce content in the alloy. 

### 3.3. Bulk Phase Composition 

**Figure 5:**
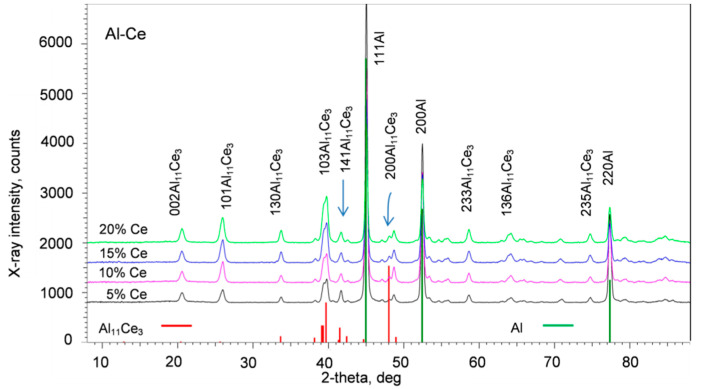
X-ray diffraction pattern of the Al–Ce alloys along with their standards for Al PDF 00-004-0787 and Al_11_Ce_3_ PDF 04-002-7472.

X-ray diffraction was used to confirm the bulk phase composition of the alloys and its change with increasing Ce content. This technique is not always reliable in the case of minor phases, since their detection is not straightforward due to low peak intensities and the superposition with strong peaks of the base. The X-ray diffraction patterns of alloys tested using a Co radiation with λ_Coα1_ = 1.78886 Å are shown in Figure 5.

Beneath the diffraction patterns, the PDF 04-002-7472 standard for the Al_11_Ce_3_ phase and the PDF 00-004-0787 standard for Al are plotted, emphasizing the strongest peaks. A comparison allowed to confirm the presence of both phases predicted from the equilibrium diagram. There is no superposition of the strongest peaks for the Al and Al_11_Ce_3_ phases. However, the strongest (200) peak for Al_11_Ce_3_ at 48.036 degrees with a theoretical intensity of 100% is absent, which could be caused by the preferred crystallographic orientation. As a result, a presence of the Al_11_Ce_3_ phase was confirmed through the second stronger peak (132) at 39.696 degrees with a theoretical intensity of 53% and a third stronger peak (033) at 39.310 deg with an intensity of 22%. An increase in peak intensity accompanying the growth of Ce in alloys reflects the increasing volume fraction of the Al_11_Ce_3_ phase of both proeutectic and eutectic origin.

### 3.4. Effect of Chemical Composition on Alloy Microstructure

**Figure 6:**
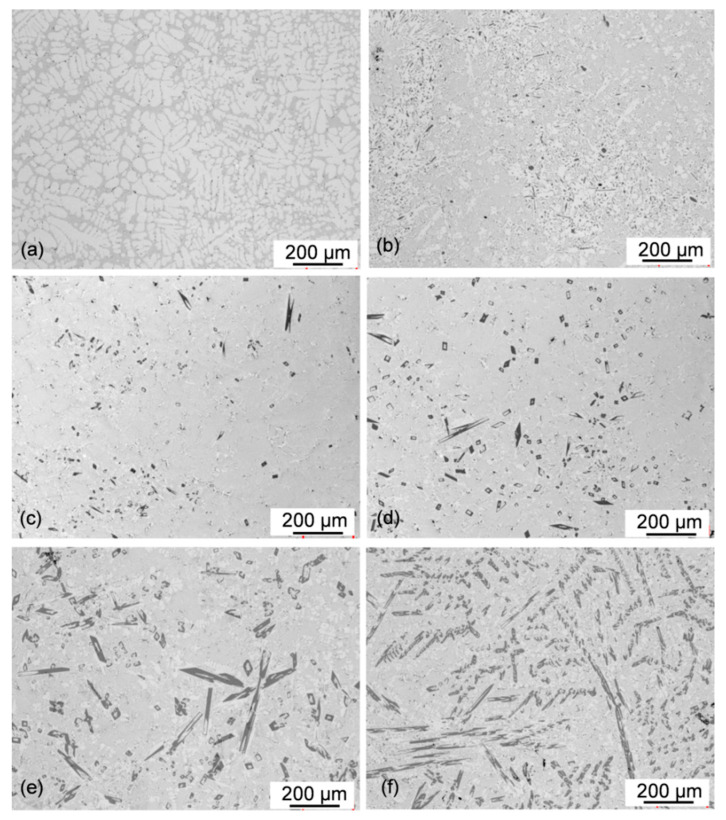
Optical micrographs showing the effect of chemical composition on the general microstructure of Al–Ce alloys: (**a**) Al–5Ce; (**b**) Al–10Ce; (**c**) Al–11Ce; (**d**) Al–12Ce; (**e**) Al–15Ce; and (**f**) Al–20Ce.

When observed with an optical microscope on unetched polished sections, the Al_11_Ce_3_ phase was seen as a dark contrast feature. The Al–5Ce alloy clearly shows the hypoeutectic nature with the proeutectic Al having dendritic morphology and occupying around half of the surface area. The eutectic is distributed in inter-dendritic regions (Figure 6a). 

Increasing the Ce content to 10 wt.% changed the alloy microstructure, causing the complete disappearance of the proeutectic Al dendrites (Figure 6b). There is some presence of fine regions with a white contrast indicating Al, uniformly distributed within the alloy microstructure. This feature represents the Al fine grains and is also present in all hypereutectic alloys. At the same time, there are also fine compounds with dark contrast, typical for the Al_11_Ce_3_ phase. A slight increase in Ce content to 11 wt.%, definitely shifted the microstructure towards the hypereutectic range with a small volume fraction of the proeutectic Al_11_Ce_3_ phase (Figure 6c). A further increase in Ce content to 12 wt.%, enlarged the content of the proeutectic Al_11_Ce_3_ phase (Figure 6d). The same direction of changes was seen for the Al–15Ce alloy with a substantial content of the proeutectic Al_11_Ce_3_ compound (Figure 6e). The contribution of the proeutectic Al_11_Ce_3_ phase increased substantially for Al–20Ce, uniformly covering the entire sample surface (Figure 6f). Thus, based on metallographic assessment, the Al–10Ce alloy with 10.61 wt.% Ce is the closest one to the eutectic composition.

For casting, the imaging of relatively large areas is essential to verify the microstructural homogeneity across the ingot surface. It allows detecting a presence of the macro-segregation of alloying elements that may affect the correlation of chemical composition with alloy microstructure. As seen in Figure 6a–f, there was no substantial macro-segregation of chemical composition. Moreover, as verified on the ingot cross section, there was no evident microstructural alignment with the solidification direction along the ingot radius.

### 3.5. Morphology of As-Solidified Eutectic and Its Change with Ce Content

Intermediate magnifications allow distinguishing the general morphology of individual phases and at the same time, relate them to the surrounding microstructure. When imaging with SEM, there is a reverse contrast to that experienced earlier in the optical microscope: the Al_11_Ce_3_ phase is seen here as the white contrast feature.

**Figure 7:**
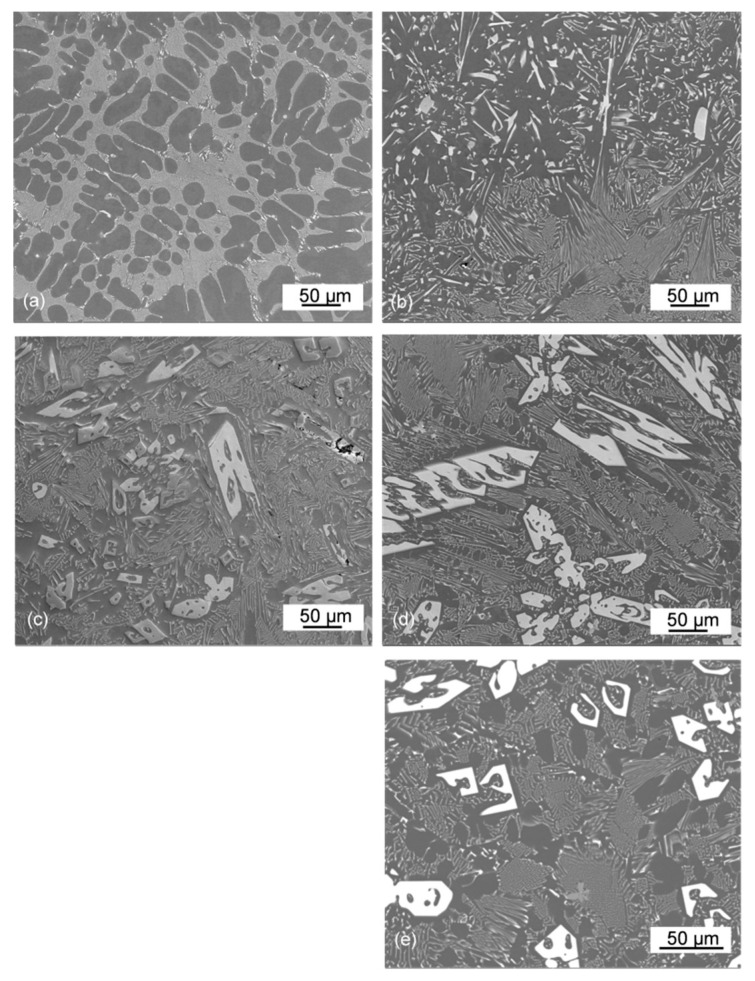
SEM images of the microstructural components in Al–Ce alloys: (**a**) Al–5Ce; (**b**) Al–10Ce; (**c**) Al–15Ce; and (**d**,**e**) Al–20Ce. The white contrast phase represents the eutectic and proeutectic Al_11_Ce_3_.

The hypoeutectic microstructure of the Al–5Ce alloy was formed by the dendritic growth of the proeutectic Al phase followed by eutectic solidification (Figure 7a). For this chemistry, the eutectic morphology was the closest one to the periodically arranged lamellae of Al_11_Ce_3_. The regularity of eutectic morphology was less pronounced for the Al–10Ce alloy with the fully eutectic composition. Although the predominant portion still represents lamellar features, as seen in the bottom right corner of Figure 7b, there is also a morphology where the Al_11_Ce_3_ phase is blocky with larger inter-Al_11_Ce_3_ distances, present in the upper side of the image. This type of eutectic is described in the literature as anomalous and is typically observed for the Al–Si alloys.

In a hypereutectic microstructure, the proeutectic Al_11_Ce_3_ phase was seen as truncated rods with a length reaching over 200 µm (Figure 7c). In addition to the parallel arrangement, some proeutectic Al_11_Ce_3_ compounds were inclined to each other. An arrangement of the individual rods suggests a certain crystallographic orientation relationship between the matrix and the proeutectic Al_11_Ce_3_ phase. This arrangement is particularly clear for the Al–20Ce alloy (Figure 7d). Imaging the different cross sections of the Al_11_Ce_3_ proeutectic compound shows its geometric nature as an assembly of lamellae into “L” or “U” shape features (Figure 7e).

**Figure 8:**
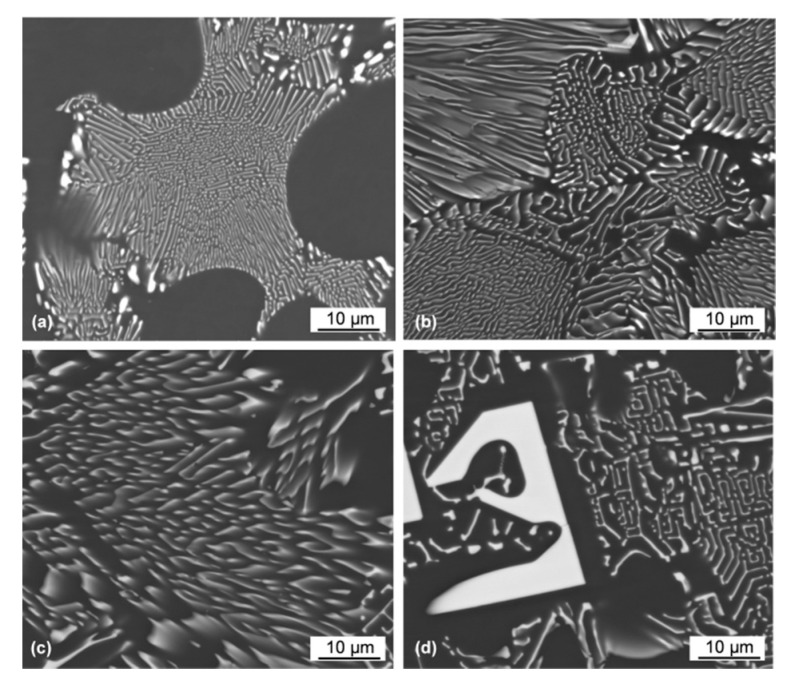
SEM magnified views of morphological changes of the Al_11_Ce_3_ eutectic phase with Ce content in binary Al–Ce alloys: (**a**) Al–5Ce; (**b**) Al–10Ce; (**c**) Al–15Ce; and (**d**) Al–20Ce.

**Figure 9:**
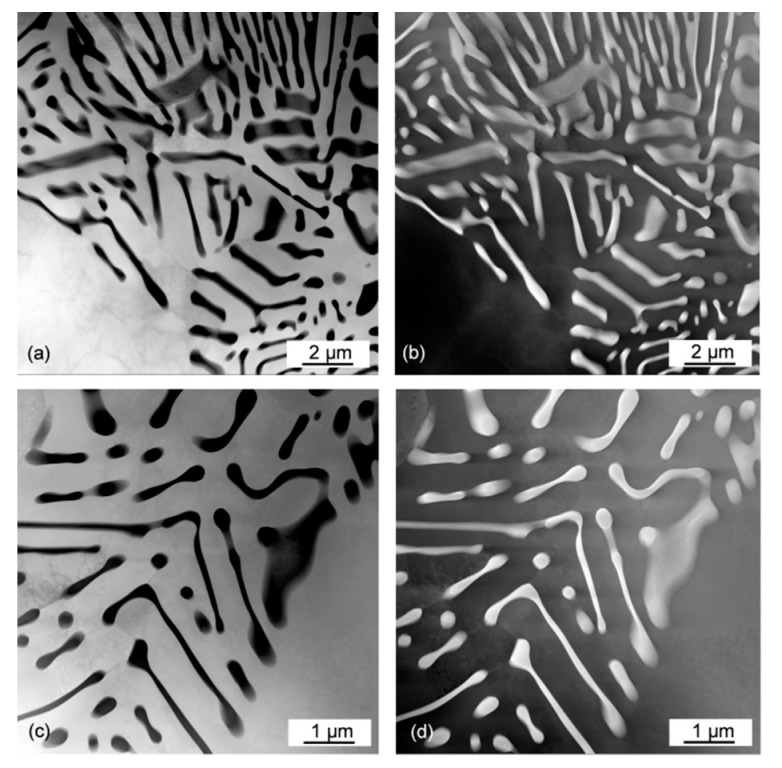
Morphology of the Al_11_Ce_3_ eutectic phase in the Al–5Ce alloy imaged by STEM: (**a**) bright field image showing the eutectic colony along with proeutectic ferrite; (**b**) HAADF image; (**c**) bright field magnified view showing a mixture of the presence of regular lamellae, rods and L-shape plates; and (**d**) HAADF image.

High-magnification imaging of the eutectic morphology in the Al–Ce alloys indicates its change with the Ce content. The regular lamellae in the hypoeutectic alloy (Figure 8a) changed their shape when moving to the fully eutectic composition of the Al–10Ce alloy. This is seen in Figure 8b, where differently oriented colonies intersect the polished section surface. The larger morphological change takes place, however, during the transient to hypereutectic compositions. As depicted in Figure 8c, some lamellae are replaced with L-shape then C-type and further with U-type shapes. It appears that there is some similarity between the morphology of the proeutectic Al_11_Ce_3_ phase and the Al_11_Ce_3_ eutectic phase with the former being more massive (Figure 8d). The surface observations of the eutectic morphology were verified through high magnification imaging using transmission electron microscopy. An example of the Al–5Ce alloy is shown in Figure 9a–d through two imaging techniques (BF and HAADF) and two magnifications. The HAADF images contain chemical information since at higher angles the scattering is related to the atomic Z-number. In addition to typical lamellae, there is some fraction of narrow lamellae evolving towards rods along with the aforementioned L-shape features.

**Figure 10:**
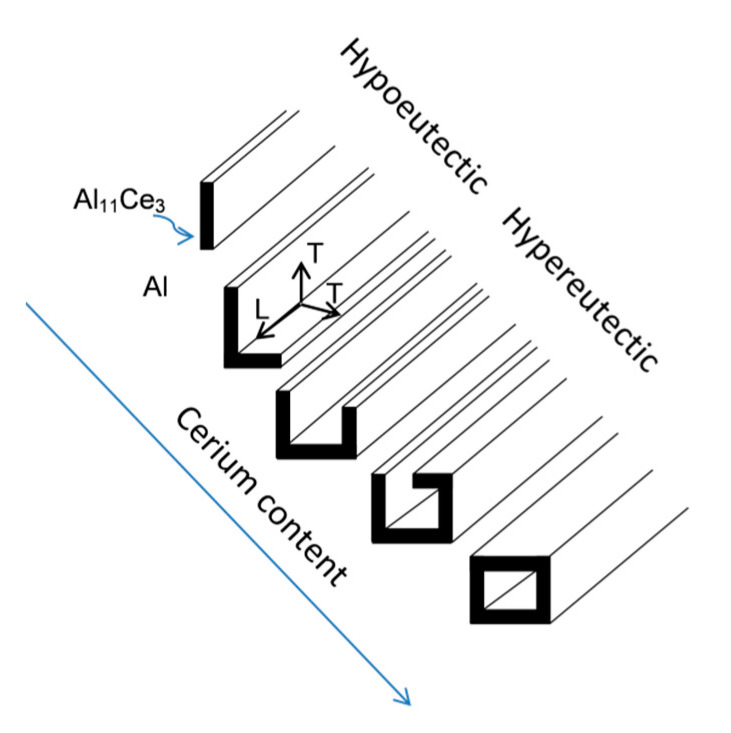
Schematic showing the effect of Ce content on morphology of the Al_11_Ce_3_ eutectic phase. The lateral growth direction is marked with L and the transverse growth direction is marked with T.

The morphological changes of the Al_11_Ce_3_ eutectic phase with the Ce content in the Al–Ce alloys, deduced from all microscopic observations, are depicted schematically in Figure 10. To form such morphologies, in addition to lateral growth as indicated in Figure 10, the transverse growth would also be required. It should be emphasized that there was no sharp change of eutectic morphology in different alloys examined and although some features became dominant, they coexisted with other shapes. Thus, for the Al–5Ce alloy, in addition to lamellae, some L–shapes and rods were also present.

### 3.6. Chemistry and Crystallography of Eutectic Phases

**Figure 11:**
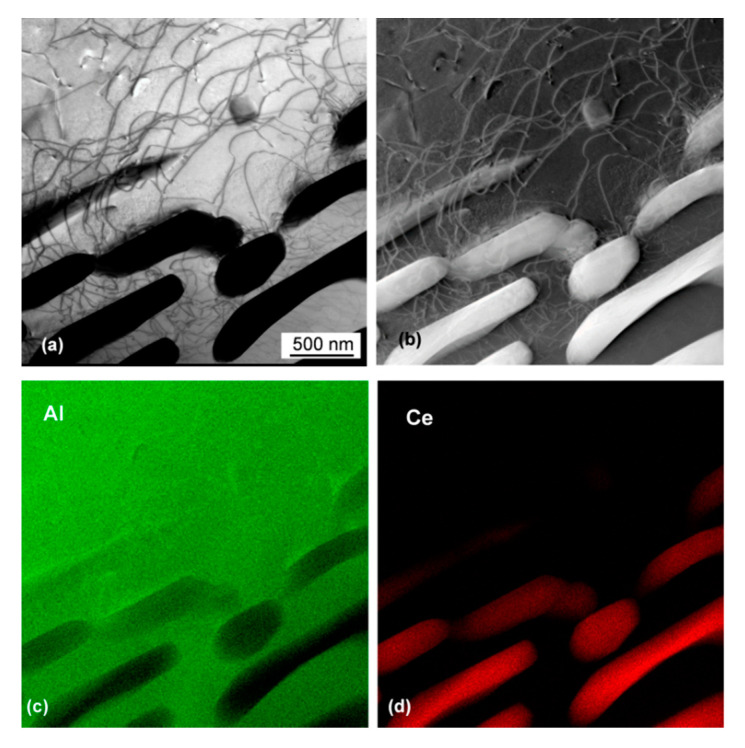
TEM view of the lamellar eutectic in the Al–5Ce alloy: (**a**) bright field image; (**b**) HAADF image; (**c**) EDS map showing distribution of Al; and (**d**) EDS map of Ce. Alloy: Al–5Ce.

The high magnification image of the lamellar eutectic is shown in Figure 11. The Al_11_Ce_3_ lamellae with a thickness of 250–400 nm are separated by an Al matrix with a thickness of 200–1000 nm. A presence of dislocations in Al between the Al_11_Ce_3_ lamellae is an indicator of high stresses generated during the solidification of this two-phase mixture. 

**Figure 12:**
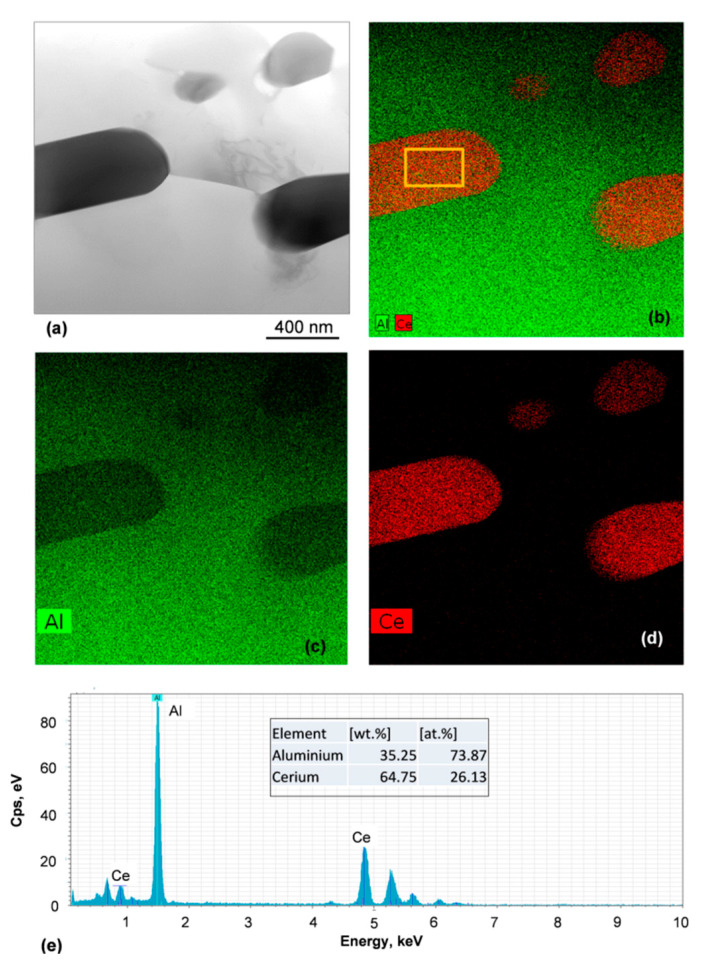
Chemical characterization of the Al_11_Ce_3_ lamellae: (**a**) bright field TEM image; (**b**–**d**) distribution map of Al and Ce; (**e**) EDS quantification of lamella composition from the area shown in (**b**) Alloy: Al–5Ce.

According to the EDS quantification, the ratio of Al/Ce is 2.83, which is below 3.66, expected for Al_11_Ce_3_ (Figure 12). This discrepancy is not clear since the reduced Al content contradicts the expected error that could be caused by the small size of lamellae and pick up of some Al signal from the surrounding Al matrix. Hence, the microchemical analysis alone does not allow for the Al_11_Ce_3_ identification.

**Figure 13:**
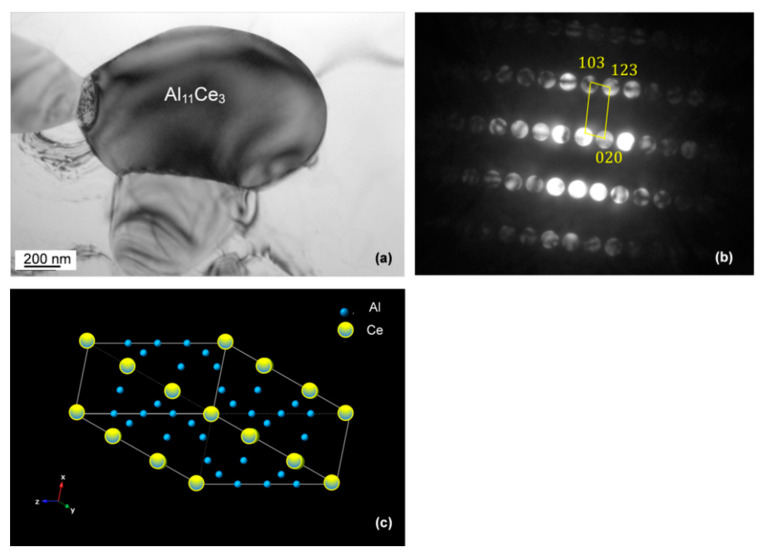
TEM bright field image (**a**) and the accompanied CBED pattern (**b**) showing the crystallographic identification of the Al_11_Ce_3_ eutectic phase. The crystal structure of Al_11_Ce_3_ is shown in (**c**). Alloy: Al–5Ce.

Although the bulk presence of Al_11_Ce_3_ was confirmed by X-ray diffraction, its individual crystallographic identification was essential. The phase with a dark contrast, shown in bright field TEM image in Figure 13a, was examined by electron diffraction. As indicated by the CBED pattern in Figure 13b, it corresponds to the αAl_11_Ce_3_ phase with a orthorhombic structure (a = 4.392 Å, b = 10.082 Å and c = 13.025 Å) with the Pearson symbol *oI28* and a space group *Immm* [25]. Its crystal structure with an arrangement of Al and Ce atoms is shown in Figure 13c. 

According to Reference [11] the αAl_11_Ce_3_ phase exists at lower temperatures and at 1006 °C it is replaced through the polymorphic transformation with βAl_11_Ce_3_, having the *tI10* symmetry. Since the same αAl_11_Ce_3_ phase exists as the proeutectic (primary) phase, commonly marked with “α” and as the eutectic phase, which should be written without “α”, to avoid confusion, in this paper the αAl_11_Ce_3_ phase is expressed simply as Al_11_Ce_3_. The αAl_11_Ce_3_ notation was used only if there was a need to distinguish it from βAl_11_Ce_3_. As explained earlier in Table 1, according to some literature sources [17,19,20] the αAl_11_Ce_3_ phase with *oI28* structure does not transform at high temperatures to βAl_11_Ce_3_ but rather to Al_4_Ce with *tI10* structure. Since Al_4_Ce has a different chemistry than αAl_11_Ce_3_, no polymorphic transformation takes place in this case. 

### 3.7. Orientation Relationship and Interface Structure between Al_11_Ce_3_ and Al within the Eutectic

High resolution transmission electron microscopy was extensively used to assess the crystallographic orientation relationship between Al_11_Ce_3_ and Al within the eutectic and the structure of the interface between them. The key objective was to verify the existence of coherency between the Al_11_Ce_3_ and Al eutectic phases. The examination was focused on the lamellar eutectic morphology, present in the Al–5 wt.%Ce alloy and its development during solidification. This was achieved through the observations of lamellae at different stages of their growth with particular attention paid to very early stages.

**Figure 14:**
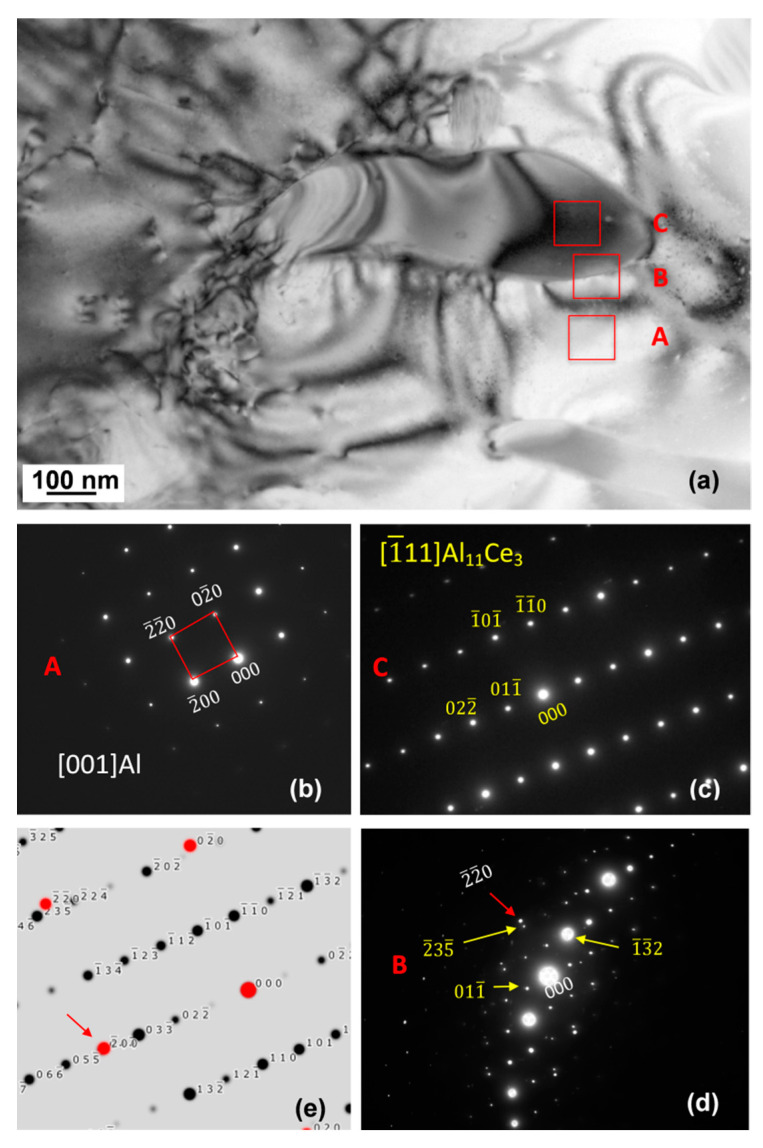
TEM bright field image (**a**) and SAD patterns of the eutectic phases at the early growth stage: Al (**b**); Al_11_Ce_3_ (**c**); interface region (**d**); simulated diffraction pattern from the interface (**e**). Orientation relationship is close to [0 0 1]_Al_ ║ $\left[\bar{1}\;1\;1\right]$_Al11Ce3_ and $\left(0\;4\;\bar{4}\right)$_Al_ ║ $\left(\bar{2}\;0\;0\right)$_Al11Ce3_. Alloy: Al–5Ce.

The TEM BF image of Al_11_Ce_3_ and Al within the eutectic is shown in Figure 14a. A contrast of the Al phase indicates subgrains, likely formed by stresses accumulated during solidification and solid-state cooling. As a possible cause, thermal expansion differences between Al and Al_11_Ce_3_ are quoted in the literature. The SAD patterns in Figure 14b–d revealed the [0 0 1]_Al_ and $\left[\bar{1}\;1\;1\right]$_Al11Ce3_ zone axes for Al and Al_11_Ce_3_, accordingly. The orientation seen in SAD in Figure 14d was taken at the interface, where both Al and Al_11_Ce_3_ are tilted away from their closest zone axes, which is [001] for Al and $\left[\bar{1}\;1\;1\right]$ for Al_11_Ce_3_. It should be emphasized that the [0 0 1]_Al_ and $\left[\bar{1}\;1\;1\right]$_Al11Ce3_ zone axes are not completely aligned, so the sample was tilted slightly after acquiring the first SAD pattern. As indicated by the SAD pattern in Figure 14d, taken from the interface region, a certain misalignment exists between the Al and Al_11_Ce_3_ lattices. It should be noted that this diffraction pattern has been taken at a much lower magnification of the bright field image in Figure 14a. Because of the strain induced by the mismatch, there is an orientation difference between the localized areas of the bright field image. While the general SAD diffraction pattern shows the average orientation of the entire area, to extract the orientation relationship between Al and Al_11_Ce_3_, the local orientations, shown in the diffraction patterns in 14b and 14c, were studied.

The simulated pattern in Figure 14e shows the superposition of the $\left[\bar{1}\;1\;1\right]$_Al11Ce3_ and [001]_Al_. As mentioned earlier, the $\left[\bar{1}\;1\;1\right]$_Al11Ce3_ zone axis was obtained by tilting just a few degrees away from the orientation shown in Figure 14d. The [001]_Al_ zone axis was also achieved by tilting only a few degrees away from the orientation shown in Figure 14d. It is believed that this orientation relationship was holding at the stage of nucleation and early growth of the Al_11_Ce_3_ lamella. As the Al_11_Ce_3_ eutectic phase grows larger and becomes incoherent with the Al matrix, the mismatch strain builds up so the Al and Al_11_Ce_3_ phases tilt further away from their initial orientation relationship. Although challenging in documentation, this phenomenon is seen as evidence that the initial orientation relationship does not hold at the advanced growth stages of the eutectic.

**Figure 15:**
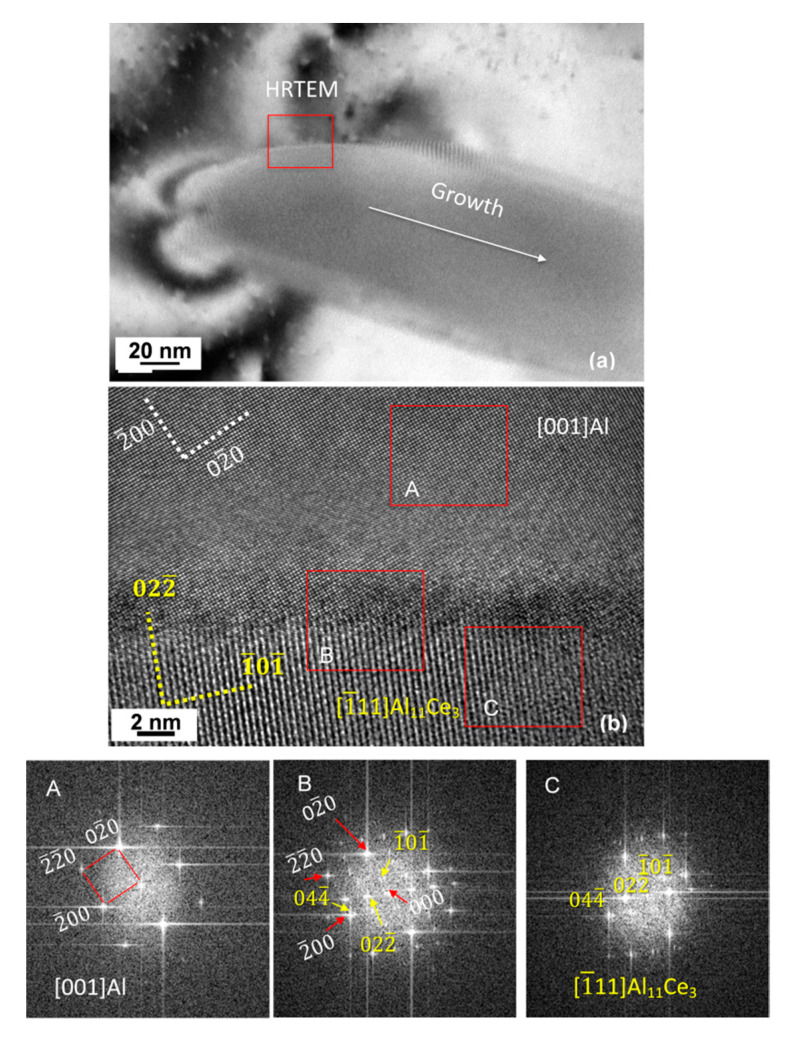
Structure of the Al_11_Ce_3_/Al interface within the eutectic during early stages of growth: (**a**) TEM bright field image with the marked growth direction; (**b**) HRTEM image of the area marked in (**a**) showing a change in coherency during growth of eutectic phases; FFT patterns from the regions marked in (**b**) for Al (A), Al_11_Ce_3_/Al interface (B) and Al_11_Ce_3_ (C). The orientation relationship is close to [0 0 1]_Al_ ║ $\left[\bar{1}\;1\;1\right]$_Al11Ce3_ and $\left(0\;4\;\bar{4}\right)$_Al_ ║ $\left(\bar{2}\;0\;0\right)$_Al11Ce3_. Alloy: Al–5Ce.

The early stages of the eutectic solidification are detailed further in Figure 15 with the Al_11_Ce_3_ phase located in the image center. Note that these HRTEM images have been taken at the exact same location as the image in Figure 14, and all the orientations discussed based on the diffraction patterns in Figure 14 apply to this image as well. The data from both the diffraction patterns and HRTEM were combined to deduce the initial orientation relationship that existed between the two eutectic phases. At the beginning of the eutectic growth, the small Al_11_Ce_3_ nuclei showed coherency but it was a common observation that the lattice misalignment between Al_11_Ce_3_ and Al was increasing during the Al_11_Ce_3_ growth. 

As shown by the FFT pattern from the interface, marked as B, the $\left[\bar{1}\;1\;1\right]$_Al11Ce3_ has an angle to [001]_Al_—suggesting that the Al_11_Ce_3_ phase rotates from its original orientation relationship with the Al matrix, as it grows. Since the FFT patterns are not very clear to deduce the orientation relationship from, the SAD diffraction was used in parallel. The FFT and SAD patterns point to the same crystals at the same zone axes, however, the SAD patterns are rotated with regard to the images and hence the FFTs are rotated with regard to the SAD pattern. An observation of the Al_11_Ce_3_/Al interface, recorded in Figure 15b, shows a worsening of the alignment between both lattices as the growth front progresses (the growth direction is marked along the interface with an arrow). In the HRTEM, the Al matrix is exactly on the [001] zone axis; therefore, the Al_11_Ce_3_ is even farther from $\left[\bar{1}\;1\;1\right]$ than what was captured in the diffraction pattern in Figure 14d. This is why the FFT of the Al_11_Ce_3_ phase does not show clear spots. Note that the Al_11_Ce_3_ orientation and hence, the Al_11_Ce_3_–Al orientation relationship is the same as that described in Figure 14. The interface, showing quite good alignment between lattices at the beginning of growth (left side of the image) is getting more and more defective as growth proceeded with the coherency dislocations formed to accommodate the lattice misfit (right side of the image). It was deduced that at the beginning of growth the orientation relationship expressed as [0 0 1]_Al_ ║ $\left[\bar{1}\;1\;1\right]$_Al11Ce3_ with (0 4 4)_Al_ ║ $\left(\bar{2}\;0\;0\right)$_Al11Ce3_ accompanied the coherency of both eutectic phases. During further growth, this orientation relationship was relaxed, so some tilt was necessary to align both zone axes.

**Figure 16:**
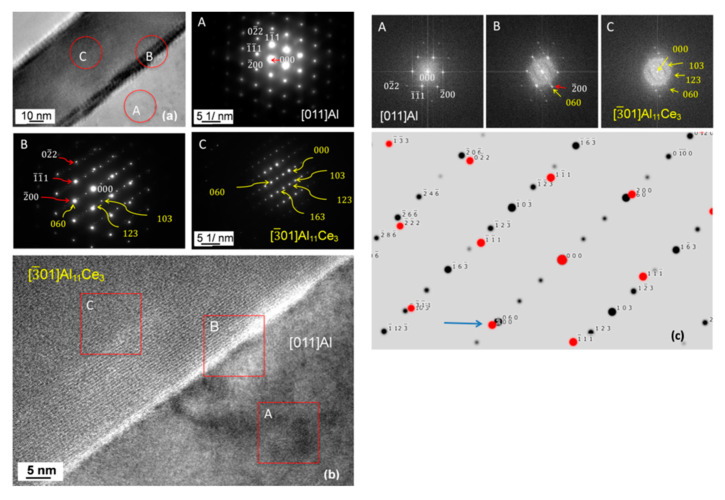
Structure of the interface between the Al_11_Ce_3_ lamella and Al having an orientation relationship: [0 1 1]_Al_ ║ $\left[\bar{3}\;0\;1\right]$_Al11Ce3_ with $\left(\bar{2}\;0\;0\right)$_Al_ ║ (0 6 0)_Al11Ce3_: (**a**) TEM bright field image and SAD patterns from Al (A), interface (B) and Al_11_Ce_3_ (C); (**b**) HRTEM image of the interface with corresponding FFT patterns A, B, C from individual phases and interface region; (**c**) simulated diffraction pattern showing an orientation relationship between Al and Al_11_Ce_3_. Alloy: Al–5Ce.

The crystallographic analysis of the orientation relationship between Al_11_Ce_3_ and Al through the SAD and FFT techniques is shown in Figure 16. An example of the interface within the lamellar structure is shown in Figure 16a. The SAD pattern for Al_11_Ce_3_ indicates $\left[\bar{3}\;0\;1\right]\;$ zone axis (C) and for Al [0 1 1]  zone axis (A). The SAD pattern from the interface, marked as (B), provides direct proof of the orientation relationship [0 1 1]_Al_ ║ $\left[\bar{3}\;0\;1\right]$_Al11Ce3_ with $\left(\bar{2}\;0\;0\right)$_Al_ ║ (0 6 0)_Al11Ce3_. The same conclusion was reached through HRTEM imaging and FFT patterns in A, B and C, as shown in Figure 16b. Similarly, as it was observed for another orientation relationship discussed earlier in this section, at advanced stages of growth, when the lamellae were well developed, the coherency was completely lost.

**Figure 17:**
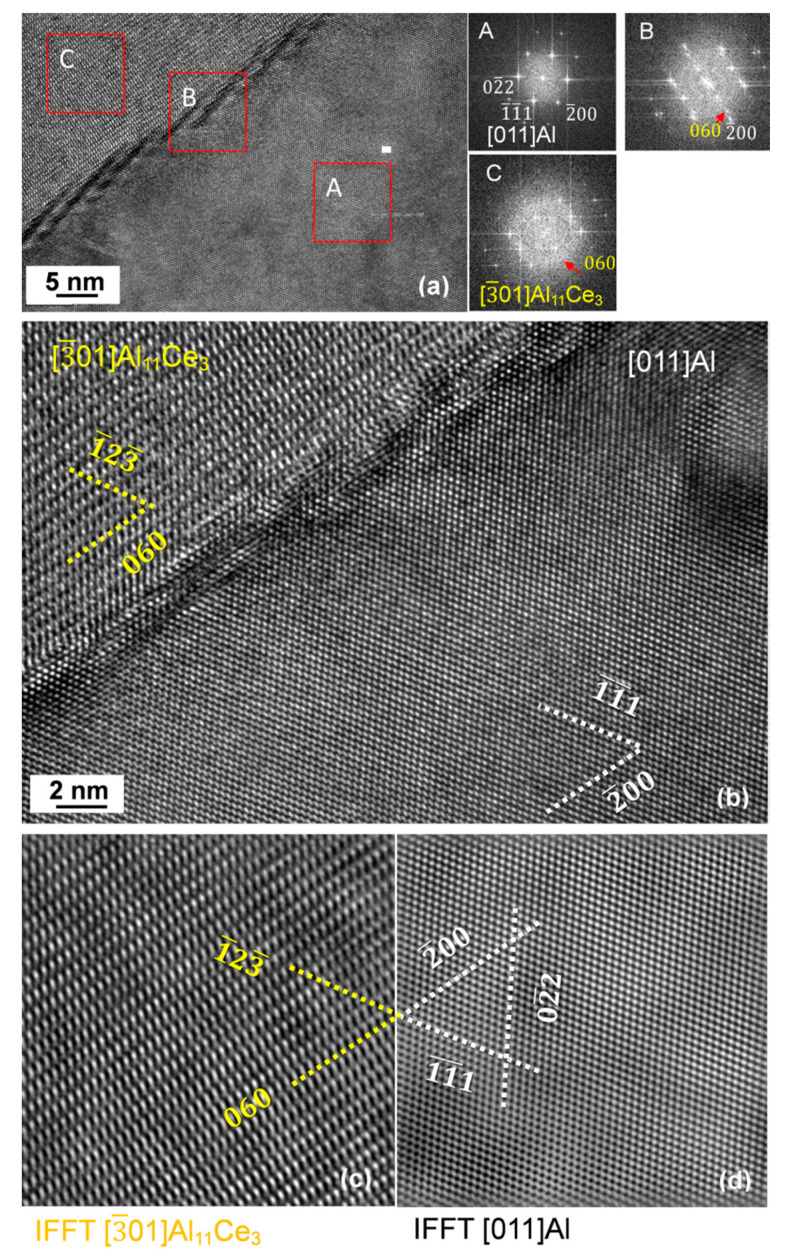
HRTEM images showing the structure of the Al_11_Ce_3_/Al interface within the eutectic: (**a**) interface with steps of high strain fields along with FFT patterns from the areas indicated; (**b**) fragment of the interface with atomic arrangements; (**c**) inverse fast Fourier transform image of the $\left[\bar{3}\;0\;1\right]$ zone axis of Al_11_Ce_3_; (**d**) inverse fast Fourier transform image of the [011] zone axis of Al. Orientation relationship: [0 1 1]_Al_ ║ $\left[\bar{3}\;0\;1\right]$_Al11Ce3_ with $\left(\bar{2}\;0\;0\right)$_Al_ ║ (0 6 0)_Al11Ce3._ Alloy: Al–5Ce.

A detailed atomic structure of the Al_11_Ce_3_ and Al interface in the eutectic during the early growth stage is shown in Figure 17. The FFT pattern revealed [0 1 1]_Al_ and $\left[\bar{3}\;0\;1\right]$_Al11Ce3_ axes indicated the orientation relationship of [0 1 1]_Al_ ║ $\left[\bar{3}\;0\;1\right]$_Al11Ce3_ with $\left(\bar{2}\;0\;0\right)$_Al_ ║ (0 6 0)_Al11Ce3_. The Al_11_Ce_3_/Al interface developed the atomic scale facets, related to the interfacial lattice matching between both eutectic phases (Figure 17a). At higher magnification, strain fields are visible which were formed due to the lattice misfit at the interface (Figure 17b). Examples of atomic structures for the $\left[\bar{3}\;0\;1\right]$_Al11Ce3_ and [0 1 1]_Al_ zone axes, generated through IFFT images, are shown in Figure 17c,d. A comparison of both structures emphasizes the atomic arrangements necessary to be created to align both lattices at the interface within the Al_11_Ce_3_–Al eutectic. 

**Figure 18:**
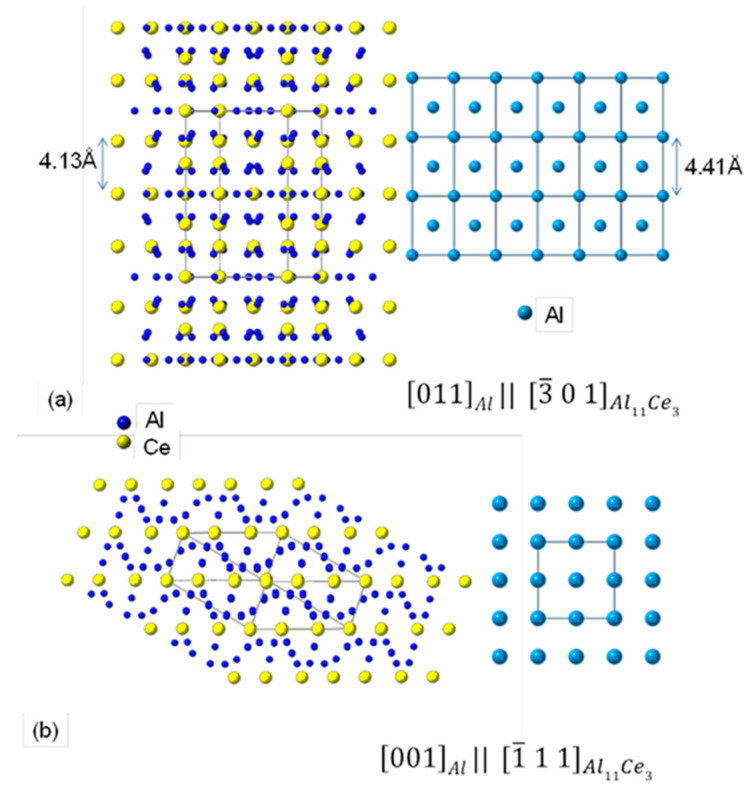
Projections of the Al_11_Ce_3_ and Al crystal structures showing the [0 1 1]_Al_ ║ $\left[\bar{3}\;0\;1\right]$_Al11Ce3_ (**a**) and [0 0 1]_Al_ ║ $\left[\bar{1}\;1\;1\right]$_Al11Ce3_ (**b**) orientation relationships. Images were modelled with the CrystalMaker^®^ software.

For the two orientation relationships identified in this study, an arrangement of atoms for both eutectic phases was generated through modelling using the CrystalMaker^®^ software. An arrangement of atoms for [0 1 1]_Al_ and $\left[\bar{3}\;0\;1\right]$_Al11Ce3_ is shown in Figure 18a and for [0 0 1]_Al_ with $\left[\bar{1}\;1\;1\right]$_Al11Ce3_ in Figure 18b. Combining these images helps to understand the complex structure of the interface between Al_11_Ce_3_ and Al within eutectic.

### 3.8. Hardness of Alloys, Eutectic and Individual Phases

**Figure 19:**
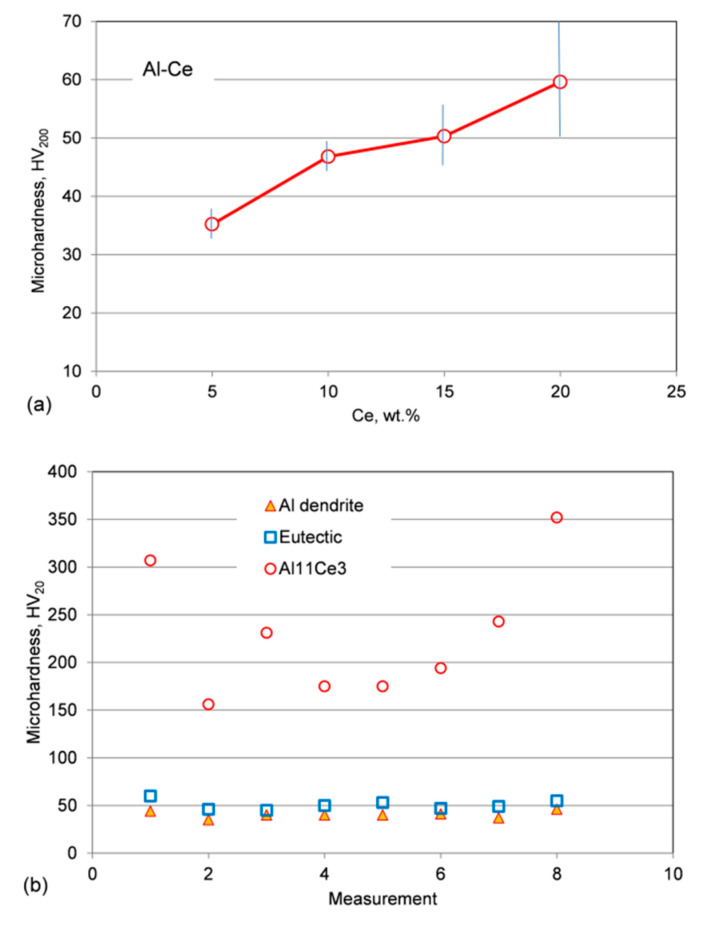
Microhardness of the Al–Ce alloys and their microstructural components: (**a**) effect of Ce content on the average microhardness of as-cast Al–Ce alloys; and (**b**) microhardness scatter of Al dendrites, the Al_11_Ce_3_ phase and the Al–Al_11_Ce_3_ eutectic.

To measure an average hardness of alloys, a load of 200 gf was selected. As verified by microscopic observations, for this load an imprint of the tester pyramidal indenter on the polished surface reached diagonal dimensions between 70 µm and 100 µm. When compared with the alloy microstructure, it confirms that the indenter covered all the microstructural components. In general, the hardness increased with Ce content (Figure 19a). The hardness of the as-cast Al–5Ce alloy reached 35 HV, which is just above the level of commercial Al, reported typically as 20–25 HV. Increasing the Ce content to 10 wt.%, eliminated the proeutectic Al and the alloy hardness reached 46 HV. Further increasing the Ce content to 15 wt.% and 20 wt.% resulted in a hardness increase to 50 HV and 59 HV, respectively. Moreover, for hypereutectic alloys the hardness data exhibited the high scatter of results with a standard deviation increased by tenfold. The higher hardness non-uniformity was caused by the presence of blocky compounds of the proeutectic Al_11_Ce_3_ phase.

To determine the hardness of individual phases, measurements were conducted under a load of 20 gf, i.e., one order of magnitude lower than the load used in previous tests. Examples of hardness readings are shown in Figure 19b. There was a scatter of results, caused primarily by the small size of the Al_11_Ce_3_ phase, so even for such a small load the indenter imprint reached the surrounding Al phase. Another factor contributing to the reading scatter was the brittle nature of the Al_11_Ce_3_ phase and its cracking under indenter load. Thus, after rejecting the abnormal data, measurements led to the Al_11_Ce_3_ hardness of 350 HV.

As seen in the same Figure 19b, the hardness of Al dendrites is one order of magnitude lower than that of Al_11_Ce_3_. At the same time, the hardness of eutectic is not substantially higher than that of Al dendrites. Such a low hardness difference suggests that the Al_11_Ce_3_ eutectic phase has a low contribution to strengthening of the Al matrix and therefore, the hardness indenter penetrated rather easily the lamellar eutectic structure.

### 3.9. Assessing Alloy Strengthening by Ce and Eutectic

**Figure 20:**
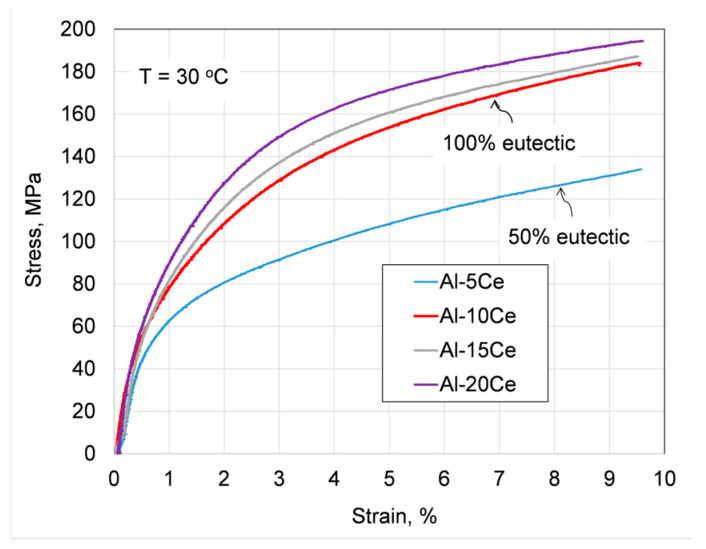
The engineering stress–strain plot for the room temperature compression of the as-cast Al–Ce alloys, showing the effect of Ce content. Yield stress determined: Al–5Ce–σ_0.2_ = 53 MPa; Al–10Ce–σ_0.2_ = 71 MPa; Al–15Ce–σ_0.2_ = 80 MPa; Al–20Ce–σ_0.2_ = 89 MPa.

To determine alloy strengthening by the eutectic structure, a uniaxial compression test was performed. Due to the low hardness of the Al–Ce alloys, to prevent barrelling, specimens with an aspect ratio Lo/D = 1.6 were used. According to ASTM E9-09 [26] such a ratio imposes some limitations, e.g., to determine the elastic modulus. The engineering stress versus strain curves, recorded at room temperature, are plotted in Figure 20 with the yield stress σ_0.2_ determined from the measurements being listed in the figure caption. The plot shows that an increase in Ce content contributed to the alloy strengthening with the greatest increment from σ_0.2_ = 53 MPa to σ_0.2_ = 71 MPa corresponding to the Ce increase from 5 wt.% to 10 wt.% Ce. This corresponds to an increase in the eutectic contribution from 50% to 100%. At the same time, the proeutectic Al_11_Ce_3_ phase had a lower contribution to alloy strengthening with σ_0.2_ = 80 MPa for Al–15Ce and σ_0.2_ = 89 MPa for Al–20Ce.

For reference purposes, the identical compression test was performed for the A380 commercial alloy (Al–8.3Si–2.4Zn–0.2Mn wt.%) which relies on the Al–Si eutectic. A room temperature testing of the as-cast alloy, without heat treatment, resulted in a much higher value of yield stress, reaching σ_0.2_ = 160 MPa.

## 4. Discussion

### 4.1. Coordinates of the Eutectic Reaction

In order to develop commercially viable alloys, knowledge of multicomponent diagrams involving functional elements is required. The starting point is, however, the binary system of major elements. Since in a cast alloy, eutectic is the vital component providing flow during the mold filling, in case of the Al–Ce system, attention is focused on the eutectic reaction, present on the Al rich side:

L ↔ Al + αAl_11_Ce_3_ (1)

Although αAl_11_Ce_3_ is commonly confirmed as the eutectic phase in the above reaction, there is an ambiguity regarding both the composition of the eutectic point and its temperature. The coordinates published show differences in the temperature from 621 °C to 645 °C and in composition from 10 wt.% to 17.8 wt.% Ce (Figure 1b). This wide scatter is mirrored in very recent publications. The metallographic assessment in [27] provided the eutectic location at 14 wt.% Ce (3 at.% Ce). In Reference [28], 660 °C, 4 at.% Ce coordinates as the experimental value and 580 °C, 2.09 at.% Ce as the Thermo-Calc calculation are cited. Moreover, a study on the casting of the Al–5wt.% Ce alloy in [29] used 621 °C from Reference [11] as the eutectic temperature in the numerical calculations and claimed 630 °C to be above the liquidus level. 

The early Al–Ce phase diagram was based primarily on References [18,30,31]. The diagram published in Reference [15], modified in Reference [16] was then used to create the 1998 version [13]. The subsequent 2011 diagram version [11], being the latest official one, on its Al rich side, does not contain the peritectic melting of βAl_11_Ce_3_ (seen as Al_4_Ce in References [17,19,32]) into Al_2_Ce and liquid at 1235 °C present in Reference [13]. Instead, the βAl_11_Ce_3_ phase forms there congruently at 1253 °C (Figure 1a). The key change is, however, the reduced eutectic temperature to 621 °C, which was derived from the computer calculations using the modified quasichemical model in References [19,20]. However, the reliability of this finding was diminished due to the fact that during the diagram optimization, as a support of such a reduced temperature of 621 °C, the DTA measurements/CALPHAD calculations from Reference [17] and older experimental findings from Reference [18] were erroneously used. In fact, the eutectic temperature, determined in [17,18], was 641 °C and 640 °C, respectively, thus rather contradicting than supporting the outcome of that computer modeling.

**Figure 21:**
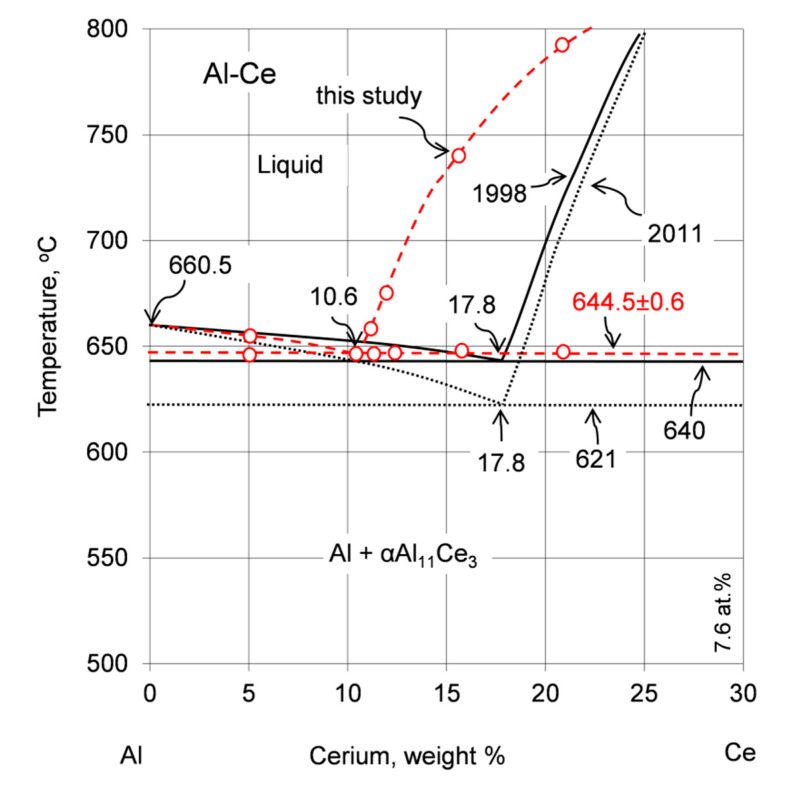
A portion of the Al–Ce phase diagram, showing coordinates of the Al–Al_11_Ce_3_ eutectic point according to two recent diagram versions: Okamoto 1998 [13] and Okamoto 2011 [11] along with results of this study.

A combination of thermal analysis and metallography in this study determined the coordinates of the eutectic transformation L ↔ Al + Al_11_Ce_3_ in the binary Al–Ce alloys at 644.5 ± 0.6 °C and 10.6 wt.% Ce, clarifying the existing literature ambiguity (Figure 21). A comparison of the cooling curves and first derivatives with plots of solid fraction versus temperature shows that a small fraction of liquid continues its transformation at temperatures below 644.5 ± 0.6 °C with solidification completed around 617–618 °C (Figure 3). It is likely a coincidence that the end of the solidification temperature determined in this study is close to the eutectic temperature obtained from modelling in References [19,20]. Software systems for the calculation of phase diagrams have been developed since the 1970s. Despite the overall progress in computer modelling, the findings of this study provide evidence that the modelling outcome still requires experimental verification before its wide implementation. It seems that the Thermo-Calc software, originally released in 1981 and used to generate the data published in 2017, led to the outcome that better matched the experiments of this study. It also appears that the statistically proven measurements in this work (Table 3) provide strong evidence that coordinates of the L ↔ Al + Al_11_Ce_3_ eutectic point in the latest Al–Ce diagram version of 2011 [11] should be revised.

### 4.2. Formation Mechanism, Morphology, and Crystallography of the Eutectic Structure

Generation of the eutectic microstructure in the Al–Ce alloys is of engineering importance because the morphology created during solidification is not subjected to the post-solidification changes and in its original form it affects the alloy properties during service. The reason is the negligible solubility of Ce in Al so the eutectic is insensitive to the post-casting heat treatment. For the same reason, heat treatment cannot be used to generate the secondary morphologies of Al_11_Ce_3_ by dissolution and precipitation processes. Thus, the only avenue to control the eutectic morphology is in a liquid state by influencing the solidification conditions [33,34]. 

During eutectic solidification, L ↔ Al + αAl_11_Ce_3_, both solid phases, form directly from the liquid; that is, locally one has L ↔ Al and L ↔ αAl_11_Ce_3_. Necessary for the reaction to proceed, a redistribution of solid, takes place in the liquid ahead of the individual interfaces, which are in close proximity. Practically all models and theories used to describe the eutectic growth process are based on the ex situ analysis of solidified microstructures. During the description of eutectic morphologies, a distinction into regular and irregular is commonly used [35,36,37]. Based on the nucleation and growth behavior of eutectic phases, the morphology depends on the entropy of solution and the relative volume of each phase [35]. The Al–Al_11_Ce_3_ eutectic morphology is characterized in the literature as mostly lamellar. Such a conclusion was reached for the Al–Ce alloys with 2.5, 4.9, and 13.8 wt.% Ce after arc melting [27] or Al–12wt.%Ce after laser melting [23]. This type of normal eutectic solidification involves two non-faceting phases, when both of them have a low entropy of fusion and morphology appears as alternate lamellae or rods within the other phase matrix. Although it might be true at the first approximation, the ex situ detailed analysis of eutectic morphologies in this study revealed more complex picture. First, imaging with both SEM and TEM/STEM shows that in addition to lamellae, there are also other morphologies within eutectics, suggesting the anomalous mechanism of its formation (Figure 7 and Figure 8). 

The eutectic structures are linked to the nature of the solid/liquid interfaces of each constituent phase. An anomalous eutectic solidification occurs when one component has a high entropy of fusion and is capable of faceting [36]. In this case, the process is sensitive to solidification conditions, resulting in a much wider range of microstructures, including broken lamellar, as irregular, complex regular or quasi regular. According to [38], the entropy conditions for the faceted growth of phase α and non-faceted growth of phase β are given as

(2) $$\frac{∆S_{f}^{\beta }}{R}\;<\;2\;<\;\frac{∆S_{f}^{\alpha }}{R}$$

where R is the universal gaseous constant, 8.314 J·mol^−1^·K^−1^. Since the entropy of fusion ∆S_f_ equals the heat of fusion ∆H_f_ divided by the melting point T_f_; $∆S_{f}=\frac{∆H_{f}}{T_{f}}$, in further considerations of this study enthalpy values are used. In the Al–Al_11_Ce_3_ system, a low enthalpy of formation for Al of 10.7 kJ/mol is accompanied by high enthalpy for Al_11_Ce_3_ as 41 kJ/mol [39] or 39.5 kJ/mol [40,41]. These values are similar as those for the Al–Si system with the enthalpy of fusion for Si being 50.2 kJ/mol [42]. The Al–Si eutectic is commonly considered as an example of anomalous reaction, typical for highly anisotropic materials. A simple calculation for Al_11_Ce_3_ (T_f_ = 1526 K) gives $\frac{∆S_{f}^{\mathrm{Al}11\mathrm{Ce}3}}{R}=3.1$ indicating the faceted growth. At the same time for Al (T_f_ = 933.5 K) $\frac{∆S_{f}^{\mathrm{Al}}}{R}=1.4$ indicates the non-faceted growth. Although there is a similarity in the enthalpies of formation, it should be pointed out that morphologies of the Al–Al_11_Ce_3_ eutectic observed here are different to those reported for the Al–Si system. The possible reason could be the material defects formed during solidification, which according to recent findings based on micro tomography, play a critical role in eutectic growth in highly anisotropic systems [42]. At the same time, for another eutectic considered as a candidate for thermally stable Al alloys, namely Al–Al_3_Ni, despite that it consists of one faceted phase (Al_3_Ni) and one non-faceted phase (Al), the product morphology is regular (rods) and resembles that of eutectic with two non-faceted phases [3].

The finding of this study is that the morphology of the eutectic depends on the Ce content in binary Al–Ce alloys (Figure 7 and Figure 8). Although for hypoeutectic compositions the structure is mostly lamellar, morphological details point towards its irregular nature. A variation in the lamella shape and in inter-lamellar spacing indicates that branching and termination occurred as a result of faceting at the solid–liquid interface. Another important finding, discovered by HRTEM, is that the faceted growth took place as well in the lamellar eutectic, as proven by the presence of the atomic size facets of the Al_11_Ce_3_ phase (Figure 17). This concludes that the L ↔ Al + αAl_11_Ce_3_ eutectic growth mechanism involved the faceted growth of the Al_11_Ce_3_ lamellae in the non-faceted Al matrix.

For the hypo- of hypereutectic Al–Ce alloys, the proeutectic phase of either Al or Al_11_Ce_3_ represents one of the two eutectic phases, so the eutectic would be expected to nucleate on the surface of the proeutectic phase, which is already solid, as the barrier to nucleation is reduced. However, this is not always the case as nucleation may depend on the barrier to nucleation of the other eutectic phase. No such phenomenon was observed in this study. This is specifically clear for the hypereutectic structure, where the proeutectic and eutectic Al_11_Ce_3_ phases are separated by a wide envelope of Al (Figure 7a, Figure 8a and Figure 9a–d). Despite the evident separation and although the proeutectic Al_11_Ce_3_ does not serve as a nucleation substrate, the morphology of the Al_11_Ce_3_ eutectic phase in hypereutectic alloys resembles the proeutectic Al_11_Ce_3_ phase (Figure 8d).

Since during eutectic solidification both eutectic phases form simultaneously, a specific crystallographic orientation relationship may develop between them, which leads to a reduction in interfacial energy. The crystallographic orientation relationship between Al_11_Ce_3_ and Al is of engineering interest because of its influence on strengthening the eutectic itself and then alloys. Some portion of this interest is created by an extraordinary strengthening effect achieved through Sc due to the presence of coherent, nano-scale, L1_2_-ordered Al_3_Sc precipitates [7]. For the Al–Al_11_Ce_3_ system, there is no consistency in the literature where different relationships are reported with a solidification rate seen as the cause of discrepancies. For the arc-melted laboratory size ingots the following orientation relationships were observed [27]: (001)_Al11Ce3_ ║ (001)_Al_, [010]_Al11Ce3_ ║ [010]_Al_, [100]_Al11Ce3_ ║ [100]_Al_. For spin casting with higher cooling rates, orientation relationships were different [34]: <001>_Al_ ║ [010]_Al11Ce3_, {020}_Al_ ║ (002)_Al11Ce3_, $\langle 3\;\bar{3}\;\bar{2}\rangle$_Al_ ║$\left[\;3\;\bar{1}\;\bar{1}\right]$_Al11Ce3_, {220}_Al_ ║ (130)_Al11Ce3_. At the same time, in the Al–10Ce–5Sr cast alloy, where most Ce was present in the eutectic, no orientation relationship was found between the Al/Al_11_Ce_3_ eutectic phases [43]. The latter is similar to the Al–Si eutectic, with Si also having a high entropy of melting with no orientation relationship between the eutectic phases [44]. 

There is generally straightforward to document the orientation relationships that exist at advanced stages of growth, including the solidification of eutectics. In this study, however, we observed that the orientation relationship Al/Al_11_Ce_3_ was only present during early stages of lamellae growth and was not maintained during further solidification. At the stage of examination, it was disappearing, which caused experimental challenges to register quality electron diffractions. Our TEM examinations led to conclusions that the exact orientation relationships were only present during lamellae nucleation. We consider this finding a novelty and through applying both the SAD and FFT we deduced the disappearing orientation relationships. The orientation relationships, identified in this study, where [0 0 1]_Al_ ║ $\left[\bar{1}\;1\;1\right]$_Al11Ce3_ with $\left(0\;4\;\bar{4}\right)$_Al_ ║ $\left(\bar{2}\;0\;0\right)$_Al11Ce3_ and [0 1 1]_Al_ ║ $\left[\bar{3}\;0\;1\right]$_Al11Ce3_ with $\left(\bar{2}\;0\;0\right)$_Al_ ║ (0 6 0)_Al11Ce3_ are different than those published so far for the Al–Al_11_Ce_3_ eutectic.

The crystallographic orientation relationship between the intermetallic phase and Al in a eutectic is related to the interfacial lattice matching between both phases. At present, there is no evidence in the literature regarding a coherency within the Al–Al_11_Ce_3_ eutectic structure. As shown in Figure 13 and Figure 18, the Al_11_Ce_3_ and Al phases have very different crystal structures. It also appears that the lamellae of the Al_11_Ce_3_ eutectic phase are simply too large to develop a coherency with the Al matrix. The role of the size factor was bypassed in the Al–Al_3_Ni eutectic, where Al_3_Ni rods with similar dimensions as the Al_11_Ce_3_ lamellae in this study, exhibited coherency with Al through a 3 nm thick shell of coherent Al formed around it [3]. The extensive HRTEM investigation in this study shed more light on the structure of the interface between Al_11_Ce_3_ and Al within the eutectic, which was found to be different than that described for Al–Si and Al–Al_3_Ni eutectics. It was deduced that for the lamellar Al/Al_11_Ce_3_ eutectic, a partial coherency existed during the nucleation and early stages of eutectic growth/solidification for the crystallographic orientation relationships specified above. However, during the growth, the Al_11_Ce_3_ eutectic phase lost its coherency with Al. A lack of coherency within the eutectic is expected to have a negative effect on the strengthening capabilities of the Al_11_Ce_3_ eutectic phase and possibly on the eutectic thermal stability.

### 4.3. Contribution of the Al_11_Ce_3_ Phase to Eutectic and Alloy Strengthening

An essential role of the Al–Al_11_Ce_3_ eutectic after solidification is seen through its potential improvement of the mechanical properties of Al–Ce alloys at increased temperatures. Due to the very low diffusion coefficient of Ce in Al [7] it is anticipated that the eutectic will improve the thermal stability of Al alloys. However, to preserve strength during the high-temperature exposures, an alloy should first achieve sufficient strength at room temperature. 

The eutectic with its highly ordered pattern can exhibit outstanding mechanical properties since its microstructure acts as natural or in situ composite [45,46]. For this reason, eutectic structures are explored in multi-principle component alloys (MPCAs) with yield strength and inter-lamellar spacing, λ, obeying a Hall-Petch-type relationship with either a λ^−1^ or λ^−1/2^ relationship [47]. In the Al–Al_11_Ce_3_ eutectic, examined in this study, the Al_11_Ce_3_ phase, having approximately 350 HV, is ten-times harder than the Al matrix with 35 HV. This unfortunately did not translate to high hardness of the Al–Al_11_Ce_3_ eutectic that reached just 45–50 HV (Figure 18). The room temperature compression test of as-cast Al–Ce binary alloys, with yield stress reaching 53–89 MPa, confirmed predictions made through hardness measurements, supporting rather limited effectiveness of the Al_11_Ce_3_ eutectic phase in overall alloy strengthening. The results are consistent with literature data, where for Al–12 wt.% Ce, the tensile yield stress is reported as 58 MPa [48]. It also appears that the strengthening achieved for the Al–Al_11_Ce_3_ eutectic is similar to that reported for the Al–Al_3_Ni eutectic [49]. The latter eutectic is seen in the literature as rather weak as compared to other binary aluminum alloys [50]. The strengthening level achieved in the Al–Ce alloys tested is well below the presently used commercial grades in similar applications, e.g., the A380 alloy, reaching under identical testing the yield stress σ_0.2_ = 160 MPa.

To achieve the strengthening level required for commercial applications, both the morphological modification of the eutectic structure and alloying with additional elements will be required. There are many conventional routes to optimize the eutectic, such as processing to create line defects [51] or degenerate lamellae into irregular structures [52]. There are also efforts to architect a dual-phase heterogeneous lamella (DPHL) structure through thermomechanical processing to feature the strength heterogeneity with soft/hard phases instead of bimodal grains [53]. Another option is morphology modification through liquid metal engineering [33]. The findings of this research are, therefore, essential for the development of multicomponent Al cast alloys, where the Al–Al_11_Ce_3_ eutectic is anticipated to provide the necessary fluidity in a liquid state and high thermal stability after solidification. 

## 5. Conclusions

A combination of thermal analysis and metallography determined the coordinates of the L ↔ Al + Al_11_Ce_3_ eutectic transformation in the binary Al–Ce alloys at 644.5 ± 0.6 °C and 10.6 wt.% Ce, clarifying the existing literature ambiguity. For a cooling rate of 0.2 °C/s, a liquid fraction of 2–10 % continued its transformation up to 28 °C below the eutectic temperature. The alloy with the exact eutectic composition exhibited the lowest liquid fraction of 2%, which solidified below the eutectic temperature, while for both the hypo- and hypereutectic compositions, that liquid fraction was higher, reaching 4–10%.

The morphology of the Al–Al_11_Ce_3_ eutectic was influenced by the Ce content in the Al–Ce alloys. Despite the high entropy of melting of the Al_11_Ce_3_ phase, in hypoeutectic alloys the eutectic was dominated by the regular morphology of periodically arranged lamellae, typical for non-faceted systems. In the lamellar eutectic, however, the faceting of Al_11_Ce_3_ was identified at the atomic scale. In contrast, for hypereutectic compositions, the Al_11_Ce_3_ eutectic phase exhibited complex morphology, transforming from lamellae towards faceted tubes/rods, requiring growth not only in lateral but also in transverse directions. A similarity was found between the morphology of the Al_11_Ce_3_ proeutectic phase and the Al_11_Ce_3_ eutectic phase, present in hypereutectic structures, suggesting that the eutectic growth was affected by the preceding solidification of the proeutectic Al_11_Ce_3_ phase.

The Al–Al_11_Ce_3_ eutectic, present in hypoeutectic alloys, contained the Al_11_Ce_3_ lamellar phase with a thickness of 200–250 nm and inter-lamellar spacing of approximately 500–1000 nm. The Al_11_Ce_3_ eutectic phase lost its coherency with Al; it was deduced that a partial coherency was present only at early stages of growth. The orientation relationships between Al_11_Ce_3_ and Al in the eutectic structure, leading to partial coherency, were determined as [0 0 1]_Al_ ║ $\left[\bar{1}\;1\;1\right]$_Al11Ce3_ with $\left(0\;4\;\bar{4}\right)$_Al_ ║ $\left(\bar{2}\;0\;0\right)$_Al11Ce3_ and [0 1 1]_Al_ ║ $\left[\bar{3}\;0\;1\right]$_Al11Ce3_ with $\left(\bar{2}\;0\;0\right)$_Al_ ║ (0 6 0)_Al11Ce3_.

The Al_11_Ce_3_ phase with a hardness of 350 HV and Al having just 35 HV in the eutectic arrangement formed an in situ composite, with the former playing a role of reinforcement. However, the coarse and mostly incoherent Al_11_Ce_3_ eutectic phase provided limited strengthening and the Al–Ce cast alloys with a fully eutectic structure reached the yield stress of just 71 MPa. This is well below the yield stress of 160 MPa, measured for the as-cast commercial A380 alloy with the Al–Si eutectic. To reach the strengthening level, required for commercial applications, a modification of the eutectic morphology through the liquid metal engineering and/or alloying with additional elements would be required.

## Figures and Tables

**Table 1:** Coordinates of the eutectic transformation L ↔ Al + Al_11_Ce_3_ in the Al-rich portion of the Al–Ce phase diagram, published within last three decades. Compositions marked with (*) were originally reported, then converted to wt.%/at.% in this paper.

Year	Temperature	Cerium Content		Eutectic Phase	Determination Method	Reference
	°C	wt.%	at.%			
1990	633	12.2	2.6	αAl_11_Ce_3_	Experimental	[12]
1998	640	17.8	4	αAl_11_Ce_3_^a)^	Based on previous experimental data in References [15,16]	[13]
2005	641	12.2	2.6	Al_11_Ce_3_^b)^	Experimental using DTA and calculated with CALPHAD	[17]
2008, 2011	621	Not specified		Al_11_Ce_3_^b)^	Optimized with modified quasichemical model and previous experimental data in References [17,18]	[19,20]
2011	621	17.8	4	αAl_11_Ce_3_^a)^	Derived based on optimized diagram in References [19,20]	[11]
2017	640, 642	Approx. 10	Approx. 2.1	Al_11_Ce_3_^b)^	Calculated with Thermo-Calc and diffusion module DICTRA in Reference [21]	[22,23]
2017	645	12	2.6	αAl_11_Ce_3_	Experimental using DSC, X-ray diffraction, metallography	[14]

^a)^—αAl_11_Ce_3_, orthorhombic, *oI28* with βAl_11_Ce_3_, tetragonal, *tI10* at high temperatures, (e.g., above 1006 °C [11] or 1020 °C [15]); ^b)^—Al_11_Ce_3,_ orthorhombic, *oI28* with Al_4_Ce, tetragonal, *tI10* at high temperatures (e.g., above 1006 °C [17] or 1002 °C [19]).

**Table 2:** Chemical compositions of the Al–Ce binary alloys investigated in this study, measured by OES/ ICP techniques.

Alloy	Ce	Si	Mg	Ni	Ti	Mn	Cu
	wt.%						
Al–5Ce	5.21 (1.05 at.%)	0.016	0.042	0.013	<0.001	<0.001	<0.005
Al–10Ce	10.61 (2.23 at.%)	0.015	0.042	0.031	<0.001	<0.001	<0.005
Al–11Ce	11.82 (2.49 at.%)	0.055	0.010	0.038	0.038	0.019	0.004
Al–12Ce	12.51 (2.68 at.%)	0.056	0.010	0.039	0.033	0.019	0.006
Al–15Ce	15.80 (3.49 at.%)	0.018	0.080	0.058	<0.024	<0.001	<0.005
Al–20Ce	20.76 (4.80 at.%)	0.017	0.110	0.072	<0.023	<0.001	<0.005

**Table 3:** Liquidus and solidus/eutectic temperatures of the Al–Ce alloys determined by thermal analysis. E_f_—end of the non-equilibrium eutectic solidification. Average eutectic temperature obtained from the measurements of all alloys equals 644.5 ± 0.6 °C.

Alloys	Liquidus, °C					Eutectic, °C					E_f_, °C
	1	2	3	Average	SDev.	1	2	3	Average	SDev.	Average
Al–5Ce	651.6	651.5	651.4	651.5	0.08	644.3	644.0	643.9	644.1	0.24	617.4
Al–10Ce				Same as eutectic		643.4	644.0	644.3	643.9	0.47	617.0
Al–11Ce	660.0	661.0	659.2	660.1	0.74	644.3	644.3	644.2	644.3	0.05	617.0
Al–12Ce	670.0	672.3	671.0	671.1	0.94	645.1	645.0	645.0	645.0	0.05	617.3
Al–15Ce	741.3	742.6	742.4	742.1	0.57	645.2	645.1	645.2	645.2	0.05	618.8
Al–20Ce	791.3	792.1	790.2	791.2	0.78	644.9	644.9	644.9	644.9	0.02	617.2
